# Evaluating the Potential Reutilizing of Fly Ash and Bottom Ash in Thailand

**Published:** 2018-06

**Authors:** Khamphe PHOUNGTHONG, Kuaanan TECHATO

**Affiliations:** 1. Environmental Assessment and Technology for Hazardous Waste Management Research Center, Faculty of Environmental Management, Prince of Songkla University, Songkhla 90112, Thailand; 2. Center of Excellence on Hazardous Substance Management (HSM), Bangkok 10330, Thailand

## Dear Editor-in-Chief

Thailand produces about 10 million tonnes of ash as solid waste which comprises of fly ash (FA), bottom ash (BA) and other incineration power plant residues (1). Since 2000, the incineration residues have been promoted in Thailand, according to the information from the Government of Thailand. “Utilization of incineration residues can provide environmental benefits by decreasing the need for landfill and reducing raw material extraction” (2, 3).

In the present work, we report the elementals concentrations of FA and BA generated from municipal solid waste incinerator (MSWI) in Thailand. Reutilizing of FA and BA, which is otherwise applications are environmental benefits (2–4). Therefore, the major aim was to investigate their use as an alternative material to replace traditional materials or natural materials with the regulatory limit values for utilization. Unfortunately, from the results of chemical compositions of FA and BA, the ash samples tracing elements of As, Ba, Cd, Cr, Cu, Pb and Zn are enriched predominantly for FA, while Ba, Cr, Cu, Hg, Ni, Pb and Zn are the most abundant elements for BA (Table 1).

**Table 1:** Comparison of chemical composition of the FA and BA samples in this study and selected references with limit values

***Elemental content(mg/kg)***	***This study***		***Ash samples***				***Limit values (7)***
	***FA***	***BA***	***FA1 (4)***	***FA2 (5)***	***BA1 (4)***	***BA2 (6)***	
Ag	ND	ND	ND	ND	ND	ND	500
As	3.74	ND	44.2	27.0	57.1	ND	500
B	ND	ND	ND	ND	ND	ND	–
Ba	7.39	114	359	405	1240	ND	10000
Be	ND	ND	6.42	6.00	1.78	ND	–
Bi	ND	ND	ND	ND	ND	ND	–
Cd	59.6	ND	1.01	ND	4.91	7.00	100
Co	ND	ND	ND	26.0	ND	ND	8000
Cr	21.5	41.7	65.8	103	330	143	500
Cu	93.9	163	56.0	34.0	1670	1128	2500
Hg	ND	936	ND	ND	ND	ND	20
Mn	ND	ND	213	ND	705	ND	–
Ni	ND	5.93	26.8	78.0	131	79.4	2000
Pb	568	46.8	86.9	ND	482	ND	1000
Se	ND	ND	16.8	13.0	1.60	ND	100
Sr	ND	ND	544	ND	219	ND	–
TI	ND	ND	ND	ND	ND	ND	700
V	ND	0.06	140	141	44.7	ND	2400
Zn	2588	269	81.9	75.0	2100	1872	5000

Note: ND is for not detectable and – not applicable.

In the BA, the constituent contents were relatively higher than in FA except for Pb and Zn. The constituents in the FA and BA were in the range of 0.06–2588 mg/kg, for heavy metals including of Ba (7.39–114 mg/kg), Cd (59.6 mg/kg), Cr (21.5–41.7 mg/kg), Cu (93.9–163 mg/kg), Ni (5.93 mg/kg), Pb (46.8–568 mg/kg) and Zn (269–2588 mg/kg). Other metals were found in minor or trace amounts and below the detection limit in the analysis for both FA and BA. The quality of ashes depended on the waste compositions and conditions of the incinerator (2–4). The contents of constituents were higher than those in the ash residues from other countries. Comparatively, Hg in BA has exceeded the regulatory limits according to the notification of the Thai standard for disposal of wastes or unusable materials (7).

Therefore the BA sample was classified as a hazardous material. BA is considered as a waste with possibility to pollute the environment and requires an appropriated disposal. Furthermore, Cd in FA was less than the limits but still high about 50 times than those from other countries. The utilization of these residues is restricted by environmental concerns in Thailand because such solid waste residues commonly contain more potentially toxic trace elements than the natural and traditional materials they replace.

Due to a large number of solid waste residues, especially FA and BA were generated by MSWI power plant and coal-fired power plant in Thailand. Many times we take these ashes were used with neglected or did not check chemical characteristics before. Of course, these hazardous elements may be leached out that could pose an environmental and human health risk posed by the residues.

Overall, there is a warning about the environmental risks resulting from FA and BA could be contaminated. Although FA and BA were suitable for the applications examined, their characteristics varied significantly depending on their site of origin. The evaluation of environmental impacts and risks associated with FA and BA needs to be performed prior to utilization of these materials. Effective environmental monitoring and protection must be carried out to ensure that FA and BA utilization and disposal does not become an environmental hazard.
